# Psychological inflexibility explains distress in parents whose children have chronic conditions

**DOI:** 10.1371/journal.pone.0201155

**Published:** 2018-07-25

**Authors:** Essi Sairanen, Päivi Lappalainen, Arto Hiltunen

**Affiliations:** 1 Karlstad University, Department of Social and Psychological Studies, Karlstad, Sweden; 2 University of Jyväskylä, Department of Psychology, Jyväskylä, Finland; Universite de Bretagne Occidentale, FRANCE

## Abstract

Experiential avoidance, cognitive defusion, and mindfulness have all been associated with psychological disorders and well-being. This study investigates whether they predict psychological distress, i.e., symptoms of burnout, depression, stress and anxiety, in parents of children with chronic conditions. We hypothesized that these factors would exhibit a large degree of common variance, and that when compared to mindfulness and defusion, experiential avoidance on its own would predict a larger proportion of unique variance. 75 parents of children with chronic conditions having burnout symptoms who participated in an intervention study completed measures of burnout, stress, anxiety, depression, experiential avoidance, cognitive defusion, and mindfulness at the beginning of the intervention study (baseline). We ran several regression analyses to assess the predictive ability of these different constructs. Experiential avoidance on its own accounted for 28–48% of the variance in different psychological symptoms. Cognitive defusion and mindfulness did not make a significant contribution to explaining burnout, stress and anxiety, but cognitive defusion contributed to explaining depression. The results confirmed our hypothesis, supporting research on the importance of psychological flexibility as a central factor in understanding the occurrence of psychological distress.

## Introduction

Parents of children with a chronic illness or functional disability have an increased risk of stress-related problems. Several studies show that such parents frequently suffer from psychological problems such as stress-related disorders, compulsive thought patterns, evasion, insecurity, despondency, fears, and reduced quality of life [1–4]. The long-term stress caused by having a chronically ill or disabled child can result in some form of chronic stress reaction and burnout syndrome, which may have serious health consequences [5–7]. Understanding of the factors that explain psychological problems in this group of parents is needed in order to develop effective treatments to support parents’ well-being.

An increasing number of research suggests that experiential avoidance (EA–attempts to avoid internal experiences) is a central trans-diagnostic process that explains a large proportion of variance in mood disorders, including parental stress [8, 9]. EA has been linked to psychological symptoms, parenting burden and stress for low-income families, and for parents of preterm infants and children with various disabilities [10–13]. Furthermore, parent EA appears to mediate the relationship between child problem behaviors and parent mental health problems in parents of children with autism spectrum disorders [12]. In addition to psychological symptoms in parents, EA has been associated with poor psychological adjustment to the child’s illness among parents of children with asthma[14].

Contextual behavior therapies such as acceptance and commitment therapy aim to affect experiential avoidance by promoting the skills of mindfulness and cognitive defusion in order to support experiential avoidance and value-related behaviors [15]. Mindfulness is a self-regulation process including attention to present-moment experiences with non-judgmental stance [16, 17]. Cognitive defusion means distancing from thoughts; as opposite to cognitive fusion, the tendency for behavior to be overly regulated and influenced by thoughts [18]. Cognitive defusion decreases believability of thoughts, and enables observing thoughts as thoughts without struggling with them as well as taking actions in contrast to thinking. Thus, the aim of the treatments is to increase psychological flexibility, defined as the ability to focus on the present moment and to regulate one’s behavior in the pursuit of goals and values [15, 19]. While there is overlap between these concepts, defusion is a more narrowly defined process that relates to mindfulness and contributes to psychological flexibility.

Current research shows that acceptance- and mindfulness-based therapies can be considered effective interventions for treating anxiety and depression [9, 20] as well as parental stress [21, 22]. In addition, several studies have demonstrated that changes in important outcomes in acceptance-based interventions are mediated by EA (e.g., [23–25]), mindfulness [26] and defusion [27], resulting in increased acceptance and flexibility. One pilot study showed that 8-week group intervention based on Acceptance and Commitment Therapy (ACT) increased parents’ psychological flexibility and later on parents’ responses to their child’s pain with parents of adolescents with chronic pain [28]. The other pilot intervention study, that combined ACT with problem-solving skills training, showed improvements in parent psychological flexibility and mindfulness and reductions in posttraumatic stress symptoms and emotional impact from their child’s life-threatening illness with parents of children who had a cancer diagnosis or life-saving cardiac surgery [29]. However, psychological flexibility related processes have been less thoroughly examined in the field of caregiving.

The objective of the current study was to investigate whether processes related to psychological flexibility, i.e, EA, defusion and mindfulness, explain psychological distress in parents of children with chronic conditions. We hypothesized that EA, defusion, and mindfulness are important predictors of psychological distress in parents of children with chronic conditions. Following the psychological flexibility model, presenting EA as a trans-diagnostic process that is central in psychopathology [19], we hypothesized that EA would have a unique incremental predictive validity over the two more specific process variables (mindfulness and defusion). We hypothesized that a) EA, mindfulness and cognitive defusion will separately predict psychological distress, b) the three processes together will predict psychological distress, and c) they will share important common variance, but d) EA will predict a larger proportion of unique variance, compared to the two other processes.

## Method

### Participants

The data is from the baseline measurements of an intervention study investigating a web-based intervention for psychological well-being among parents of children with type 1 diabetes or functional disabilities. The data was collected in two phases, first group in spring and second in fall, 2017. Participants were recruited through a hospital and a children’s and adolescents’ clinic and habilitation center of the County Council by sending an invitation letter to all potential participants in Värmland. Participants enrolled in the study via e-mail or by phone. The initial exclusion criteria were assessed through online survey including a questionnaire for reported burnout symptoms. To be eligible for this study, the participants had to have a score exceeding 2.75 points on the Shirom-Melamed Burnout Questionnaire (SMBQ, [30]), which indicates having at least low level of burnout. The level of disability in children was not evaluated as an inclusion criteria, because the focus was on parents’ wellbeing. Persons with a poor knowledge of Swedish (i.e., those who could not fill out the questionnaires in Swedish) were excluded from the study, as were those undergoing some other psychological treatment. The subjects were required to have access to the Internet and use a computer daily. A total 83 persons enrolled in the study. Of these, five were excluded: three of them did not meet the SMBQ inclusion criteria, and two did not have the time to participate. In addition, three persons did not complete the baseline measurements.

Finally, 75 participants (14 male, 61 female) completed the baseline measurements. Given that this group included 8 couples, only 67 children were represented. The mean age of the participants was 42.6 ± 6.9 years (range 28–58). 52% of the participants had a university level education. 48% of the children (N = 67) had type 1 diabetes and the others had long-term, inherent, or early psychological or physiological functional disabilities, including mostly ADHD, autism, Asperger syndrome, and cerebral palsy.

All participants gave written, informed consent to their participation in the study. The study was approved by the Regional Ethical Review Board at Uppsala University and it was performed in accordance with the Declaration of Helsinki.

### Measurements

Participants completed a web-based survey that included the following self-report measures.

### Process measures

**Experiential avoidance** was measured by the Acceptance and Action Questionnaire (AAQ-II, Bond et al., 2011). It includes 7-items that assesses the ability to accept negative emotions and other internal experiences and to take value-based actions in the presence of these experiences. The questions in the AAQ-II are based on statements like “My painful experiences and memories make it difficult for me to live a life that I would value.” The items were rated on a 7-point Likert-type scale ranging from 1 (never true) to 7 (always true), with higher scores indicating higher levels of experiential avoidance (i.e., lower levels of psychological flexibility). The structure, reliability, and validity of the AAQ-II have been supported [31].

**Mindfulness** was assessed by the Five Facet Mindfulness Questionnaire (FFMQ, Baer et al., 2006) that measure a tendency of paying attention to present moment in daily life. It includes 39-items that are rated on a 5-point Likert-type scale ranging from 1 (never or very rarely true) to 5 (very often or always true), with higher scores indicating higher levels of mindfulness. It includes the following elements. (a) *Observing* includes noticing internal and external experiences. (b) *Describing* involves naming and labeling internal experiences. (c) *Acting with awareness* means paying attentions to one’s activities of the moment. (d) *Non-judgment of inner experiences* means having a non-evaluative stance toward inner experiences. (e) *Non-reactivity to inner experiences* is the ability to let thoughts and feelings to come and go without struggling with them. The structure, reliability and validity of FFMQ have been supported [32].

**Cognitive fusion** was measured with the Cognitive Fusion Questionnaire (CFQ, [18]. It includes 13-items that are rated on a 7-point Likert-type scale ranging from 1 (never true) to 7 (always true) with higher scores indicating higher levels of cognitive fusion. The CFQ contains items reflective of the believability of thoughts, getting stuck on thoughts and taking action in contrast to thinking. The questions of the CFQ are based on statements like “I struggle with my thoughts.” The reliability and validity of CFQ have been supported [18].

### Outcome measures

**Burnout symptoms** were measured with the Shirom-Melamed Burnout Questionnaire (SMBQ, [30, 33, 34]. The SMBQ measures four elements of burnout; Emotional exhaustion and physical fatigue, Listlessness, Tension, and Cognitive weariness. The SMBQ consists of 22 items that are rated on a seven-point scale ranging from 1 'Never or almost never' to 7 'Always or almost always'. High scores correspond to more burnout symptoms. The cut-off scores for burnout on the SMBQ are 2.75–3.74 indicating low burnout, 3,75–4,46 indicating high burnout and ≥ 4.47 indicating pathological level of burnout. SMBQ’s psychometric characteristics and factorial validity have been supported [30, 33]

**Emotional states of depression, anxiety and stress** were measured with the twenty-one item Depression, Anxiety and Stress Scale (DASS-21, [35]. The DASS-21 is a self-report assessment that contains three subscales scored on a Likert four-point scale (0, 1, 2 and 3), ranging from 0 (“Strongly Disagree”) to 3 (“Totally Agree”). Each subscale of the DASS consists of seven items that evaluate the emotional states of depression, anxiety, and stress. The factor structure and validity of the DASS-21 have been supported *[36]*

The measures were written in Swedish. The measures have been translated and back translated for previous studies [33, 36–38], except the Cognitive Fusion Questionnaire (CFQ) that was translated for this study by a group of researchers with long experience in acceptance-, mindfulness-, and value-based interventions. The internal consistency of the measures and subscales was good (Cronbach’s α = .83 –.91, see Table 1).

**Table 1:** Correlations, means (M), standard deviations (SD), and Cronbach’s alphas (α) for measurements, N = 75.

	1.	2.	3.	4.	5.	6.	7.
*1. AAQ*	–						
*2. CFQ*	.74**	–					
*3. FFMQ*	-.51**	-.65**	–				
*4. SMBQ*	.53**	.47**	–.37**	–			
*5. DASS depression*	.69**	.64**	-.36**	.54**	–		
*6. DASS anxiety*	.54**	.50**	–.39**	.50**	.67**	–	
*7. DASS stress*	.60*	.56**	–.41**	.63**	.65**	.70**	–
M	22.45	49.37	117.28	4.84	6.48	4.17	9.85
SD	9.57	12.28	17.26	0.87	4.70	4.10	4.85
α	.90	.84	.86	.91	.89	.83	.85

Note. AAQ = Acceptance and Action Questionnaire, CFQ = Cognitive Fusion Questionnaire, FFMQ = Five Facet Mindfulness Questionnaire, SMBQ = Shirom-Melamed Burnout Questionnaire, DASS = Depression, Anxiety and Stress Scale.

* *p* < .05

** *p* < .01 (2-tailed)

### Statistical analysis

The statistical analyses were conducted using Mplus (Version 8) and SPSS (Version 24). Correlations between variables were calculated by using Pearson's correlations and several regression analysis were conducted to investigate the study hypothesis. First, we ran several regressions to assess the total variance (R^2^) that could be attributed individually to the three predictors (AAQ, CFQ and FFMQ). Separate regressions were run with each individual predictor for each of the four outcomes (Models 1–3, Table 2). Second, we ran several multivariate regressions with AAQ, CFQ and FFMQ as predictors and *burnout* (SMBQ), *depression* (DASS depression), *anxiety* (DASS anxiety), and *stress* (DASS stress) as outcomes to determine whether the three predictors combined predict these psychological symptoms. The multivariate regressions (Model 4) included all three predictors. Significance of predictors were calculated by using confidence intervals based on 1000 bootstrap samples. Increasing the number of samples did not affect the results. Predictors are deemed statistically significant at the .05 level, if the 95% confidence interval (CI) for the estimate of predictor does not include zero. There were no missing values in the data.

**Table 2:** Standardized estimates of predictors in linear models explaining burnout, depression, anxiety and stress, with 95% confidence intervals reported in parentheses. N = 75.

Model	Predictors	*Burnout*	*Depression*	*Anxiety*	*Stress*
1.	*AAQ*	.53* (.35, .67)	.69* (.56, .79)	.54* (.37, .66)	.60* (.43, .73)
	R^2^	.28	.48	.29	.36
2.	*CFQ*	.47* (.32, .62)	.64* (.51, .74)	.50* (.37, .62)	.56* (.40, .68)
	R^2^	.22	.41	.25	.32
3.	*FFMQ*	-.37* (-.55, -.17)	-.36* (-.53, -.18)	-.39* (-.56, -.21)	-.41* (-.56, -.25)
	R^2^	.13	.13	.15	.17
4.	*AAQ*	.39* (.09, .68)	.49* (.29, .67)	.37* (.10, .62)	.41* (.13, .64)
	*CFQ*	.14 (-.29, .47)	.36* (.11, 56)	.17 (-.09, .40)	.23 (-.01, .48)
	*FFMQ*	-.08 (-.42, .18)	.13 (-.10, .34)	-.10 (-.33, .14)	-.06 (-.24, .16)
	Total R^2^	.30	.52	.32	.40
	Common R^2^	.22	.34	.24	.30
	AAQ ^R2^	.07	.11	.06	.08
	CFQ ^R2^	.01	.05	.01	.02
	FFMQ ^R2^	.00	.02	.01	.00

Note: AAQ = Acceptance and Action Questionnaire, CFQ = Cognitive Fusion Questionnaire, FFMQ = Five Facet Mindfulness Questionnaire.

Confidence intervals based on 1000 bootstrap samples. R^2^ = proportion of outcome variable variance explained by predictor(s); ^R2^ = proportion of variance attributable only to a specific predictor.

* = Statistically significant predictor.

## Results

### Correlations

Descriptive statistics and Pearson's correlations are reported in Table 1. All three predictors, experiential avoidance (AAQ), cognitive defusion (CFQ) and mindfulness (FFMQ), correlated significantly with psychological distress–namely, burnout, anxiety, depression, and stress–and each association was in the predicted direction.

### Regression analysis

Table 2 presents the results of the simple and multivariate regression analysis. All processes were significant in explaining all outcomes (confidence intervals excluded zero), when they were investigated separately (Models 1–3). Instead, multiple regression (Model 4) indicated that the combined total scores of three predictors (AAQ, CFQ and FFMQ) significantly predicted *burnout*, *depression*, *anxiety*, and *stress*, but only AAQ was a significant predictor in all these models. In addition, CFQ was a significant predictor of depression together with AAQ.

Next, we studied the overlap in prediction between the processes and the unique contribution in variance that can be attributed to each process, above and beyond the others. Table 2 presents the part of the variance predicted (R^2^) by the three processes together, variance that is common to the three processes, and the part of variance that is unique to each of them. The AAQ predicted more unique variance than the other predictors for each of the outcomes.

## Discussion

The purpose of this study was to investigate and compare experiential avoidance, cognitive defusion and mindfulness in terms of their ability to predict psychological distress (burnout, stress, anxiety, and depression) in parents of children with chronic conditions. As expected, all these processes explained psychological distress, when they were investigated separately. Cognitive defusion (CFQ) accounted for between 22% and 41% of the variance for the different outcomes, mindfulness (FFMQ) for between 13% and 17%. Experiential avoidance (AAQ) accounted for 28% to 48% of the variance, showing its greater predictive ability.

When investigating the use of all three processes together to explain psychological distress, experiential avoidance significantly predicted all outcomes, while cognitive defusion also contributed to predicting symptoms of depression. As regards common variance, there was a large degree of overlap between the three processes. This could be expected, given that experiential avoidance, cognitive defusion, and mindfulness are all constructs related to the way we deal with our thoughts and emotions. Yet, the results revealed that experiential avoidance (AAQ) also explained 6% to 11% of the variance in psychological symptoms that was not explained by cognitive defusion and mindfulness. Whereas measures of cognitive defusion and mindfulness may assess more carefully the way we deal with our inner experiences, the AAQ also considers the effect of inner experiences on value-related behaviors, which may explain its greater predictive ability.

These results show that experiential avoidance is a robust predictor of psychological symptoms such as anxiety, depression, stress, and burnout. The results are consistent with a number of studies indicating that experiential avoidance (or its reversed form, psychological flexibility) is an essential process in psychological disorders [39]. Psychological flexibility uniquely predicts several positive therapeutic outcomes [8]. Experiential avoidance can therefore be seen as an essential factor in psychological vulnerability, and psychological flexibility as a buffer against distress, protecting individuals from developing psychological disorders.

In the present study, the general measure of EA (AAQ-II) was used, but there exist several measures that have been modified for different populations. Targeted measures of EA have found to be more accurate in predicting psychological and behavioral outcomes [23, 25, 40]. There exist also targeted measures for experiential avoidance/ psychological flexibility related to parenting such as the Parental Psychological Flexibility (PPF[41]) Questionnaire, designed to assess how parents’ of pre-adolescents and adolescents accept negative thoughts, emotions and urges about one’s child and still act in ways that are consistent with effective parenting, and the Parent Psychological Flexibility Questionnaire (PPFQ[42]) developed for parents of young people with chronic pain. In the future studies, it could be useful to include both general and targeted measures of EA in predicting psychological and behavioral outcomes in parents, because they may provide completing understanding of processes relating to parenting.

The findings should be taken in the context of certain limitations. Firstly, the cross-sectional design precludes firm causal inferences. It is possible, for instance, that lower levels of distress led participants to report being more accepting of difficult thoughts and emotions. Longitudinal and experimental studies may be needed to assess the protective power of psychological flexibility over time. Secondly, the participants were Swedish adults (mostly women) who had children with type 1 diabetes or functional disabilities; thus, the generalizability of the results is limited. Thirdly, we only used self-reported measures, and thus further research should replicate these results with behavioral or biological outcomes. In addition, in future studies, more detailed investigation are needed about how child’s characteristics (e.g., age, gender, onset and the current conditions of the chronic diseases) affect psychological flexibility and wellbeing of parents. It is possible that these background variables moderate the relationship between parents’ psychological flexibility and wellbeing.

Notwithstanding these limitations, we believe that this research advances our understanding of the processes involved in parental distress, and that some theoretical and applied conclusions can be drawn. At the theoretical level, our results suggest that many current theoretical approaches could boil down to a process of experiential acceptance, i.e., the ability to accept one's thoughts and emotions in order to enhance one's value-related actions. At a clinical level, our results add to the body of research showing that acceptance and mindfulness-based therapies can be used for caregivers [21, 22]. In interventions, it is important to bolster the distancing and acceptance of thoughts and emotions with parents whose children have chronic illness or functional disabilities. This may broaden the intervention repertoire used in family support programs to better address the specific needs of the parents.

## Supporting information

S1 File Data of the study. (SAV)
